# Associations Between Dietary Protein Sources, Plasma BCAA and Short-Chain Acylcarnitine Levels in Adults

**DOI:** 10.3390/nu11010173

**Published:** 2019-01-15

**Authors:** Michèle Rousseau, Frédéric Guénard, Véronique Garneau, Bénédicte Allam-Ndoul, Simone Lemieux, Louis Pérusse, Marie-Claude Vohl

**Affiliations:** 1 Institute of Nutrition and Functional Foods (INAF), Laval University, Quebec City, QC G1V 0A6, Canada; michele.rousseau.1@ulaval.ca (M.R.); frederic.guenard@fsaa.ulaval.ca (F.G.); veronique.garneau@fsaa.ulaval.ca (V.G.); benedicte.allam-ndoul@criucpq.ulaval.ca (B.A.-N.); simone.lemieux@fsaa.ulaval.ca (S.L.); louis.perusse@kin.ulaval.ca (L.P.); 2 School of Nutrition, Laval University, Quebec City, QC G1V 0A6, Canada; 3 Department of Kinesiology, Laval University, Quebec City, QC G1V 0A6, Canada

**Keywords:** branched-chain amino acids, acylcarnitines, dietary protein sources, meat, metabolic syndrome, metabolite profiling, diet

## Abstract

Elevated plasma branched-chain amino acids (BCAA) and C3 and C5 acylcarnitines (AC) levels observed in individuals with insulin resistance (IR) might be influenced by dietary protein intakes. This study explores the associations between dietary protein sources, plasma BCAA levels and C3 and C5 ACs in normal weight (NW) or overweight (OW) individuals with or without metabolic syndrome (MS). Data from 199 men and women aged 18–55 years with complete metabolite profile were analyzed. Associations between metabolic parameters, protein sources, plasma BCAA and AC levels were tested. OW/MS+ consumed significantly more animal protein (*p* = 0.0388) and had higher plasma BCAA levels (*p* < 0.0001) than OW/MS− or NW/MS− individuals. Plasma BCAA levels were not associated with BCAA intakes in the whole cohort, while there was a trend for an association between plasma BCAA levels and red meat or with animal protein in OW/MS+. These associations were of weak magnitude. In NW/MS− individuals, the protein sources associated with BCAA levels varied greatly with adjustment for confounders. Plasma C3 and C5 ACs were associated with plasma BCAA levels in the whole cohort (*p* < 0.0001) and in subgroups based on OW and MS status. These results suggest a modest association of meat or animal protein intakes and an association of C3 and C5 ACs with plasma BCAA levels, obesity and MS.

## 1. Introduction

Branched-chain amino acids (BCAA) are comprised of leucine, isoleucine and valine [1]. Their plasma levels have been positively associated with features of the metabolic syndrome (MS), such as insulin resistance (IR) and pre-diabetes [2,3], and thus with an increased risk of type 2 diabetes (T2D) and cardiovascular diseases (CVD) [4,5,6,7,8]. Controversies still remain on whether an increase in plasma BCAA levels is a cause or a consequence of IR. The latter is the most strongly supported hypothesis [9,10], since plasma BCAAs elevation could be the result of an impaired metabolism caused by the decreased gene expression of BCAA aminotransferase (*BCAT*) and branched-chain a-keto acid dehydrogenase (*BCKD)*, as seen in mice models [11].

Most dietary BCAAs are metabolized in the skeletal muscle after passing through systemic circulation, whereas other amino acids (AA) are metabolized in the liver [12,13,14,15]. This reinforces the potential impact of BCAAs on circulating metabolites, hormones or nutrients [15]. Some studies also relate the increase of plasma BCAAs to the amount or the type (animal or vegetal) of protein ingested [16,17]. Moreover, diets high in red meat [18], animal protein or BCAAs [8,19,20] are associated with an increased risk of T2D in contrast to diets high in vegetal protein, which appears to be associated with a lower risk of T2D [19,20]. In addition, acylcarnitines (AC), a by-product of incomplete mitochondrial fatty acid oxidation, are acyl esters of carnitine that can also result from the degradation of other compounds, such as BCAAs into C3 and C5 ACs [21]. More specifically, isoleucine and leucine catabolism generate 2-methylbutyryl-CoA and isovaleryl-CoA, which will transfer their acyl group to carnitine to form C5 ACs. Isoleucine and valine catabolism will generate propionyl-CoA to be incorporated into C3 ACs [22,23]. These short-chain ACs have previously been associated with IR [21,22] along with western-type dietary habits [24], and are considered as a potential marker of animal products and meat consumption [25].

Changes in plasma BCAA levels according to dietary profiles and dietary protein intakes have been investigated. While higher BCAA intakes have been related to plasma BCAA levels in some studies [16,26], others found no or an inverse association between these two factors [27,28,29]. One possible explanation for this discrepancy may be related to the source of protein, either animal or vegetal [4]. Protein source might also influence the relationship between plasma BCAA levels and IR. Accordingly, red meat, poultry, fish and whole milk were reported to be the main sources of dietary BCAAs in the US [8] and UK [28] populations, two countries for which a positive association between IR and plasma BCAAs has been reported. An association between IR and plasma BCAAs have also been observed in population from Brazil where red meat, poultry, bread, rice and beans were the principal dietary sources of BCAAs [30]. In contrast, cereal, potatoes and starches, followed by fish, shellfish and finally meats were the main sources of BCAAs in a Japanese cohort where an inverse relationship between BCAA intakes and T2D risk was observed, but only in women [29]. However, up to now, no study has explored the associations between the principal dietary sources of protein and plasma BCAAs, as well as its association with C3 and C5 AC levels in one single cohort. 

As such, the main objective of this study was to investigate the relationship between dietary protein source—either animal or vegetal—intakes and fasting plasma BCAA levels in adults with diverse BMI and obesity-associated metabolic perturbations. The second objective was to describe the association between plasma BCAA levels and C3 and C5 AC levels in the same subgroups according to overweight (OW) MS status. We found plasma BCAA levels to be associated with animal protein consumption, with red meat being the main source of proteins that correlates in OW/MS+ individuals. C3 and C5 plasma concentrations were also associated with plasma BCAA levels in the whole cohort, and by subgroups defined on the basis of BMI and the metabolic status. 

## 2. Materials and Methods 

### 2.1. Study Population

INFOGENE is a cross-sectional study investigating the familial history of obesity [31,32,33]. The recruitment took place in the Quebec City metropolitan area between May 2004 and March 2007 via advertisements in local newspaper and radio stations. Electronic group messages were also sent to university and hospital employees. In the first period of recruitment, only normal weight (NW) individuals were accepted while in the second phase, only OW individuals were recruited. No other criteria of exclusion were applied. After a phone interview where a trained research assistant asked the participants to report their weight and height, eligible individuals were given an appointment at the clinical investigation unit. At this appointment, anthropometric measurements were taken, and participants had to complete a food frequency questionnaire (FFQ), as well as other questionnaires assessing socio-demographic level and lifestyle habits. Individuals who were homeless (1), pregnant (1), older than 55 years (1), had acquired immune deficiency syndrome (AIDS) (1), total energy intakes greater than 4 SD (4), fibre intakes greater than 4 SD (1) or who reported unreliable data (1), were excluded. The final sample consisted of 664 adults—of which 245 men and 372 women—aged 18 to 55 years who gave their written consent to participate. Of those individuals [34], 100 men and 100 women were randomly selected for metabolic profiling of their blood samples [35]. One individual missing biochemical information was excluded from the following analyses. This study has been approved by the Université Laval Ethics Committee.

### 2.2. Dietary Assessment and Food Grouping

Dietary intakes over the past month were assessed using a 91-item FFQ administered by a registered dietitian. This FFQ was previously validated in French Canadian men and women, and was structured to reflect nutritional habits of the Quebec population [36]. Nutritional intakes were evaluated using the Nutrition Data System for Research (NDS-R) software version 4.03 (Nutrition Coordination Center, Minneapolis, MN, USA). For each item in the FFQ, participants were asked to report their consumption either in days, weeks or months. Many portion size examples were provided for a better estimation of the consumption. Thirty-seven food groups were made, based on the nutrient profile of each item or on its culinary usage. Some groups consisted of only one food (e.g., eggs or beer) because of their particular composition. Twelve groups typically providing most of the dietary proteins in Canada were kept for the current analysis [37]. These are red meat, processed meat, organ meat, fish and other seafood, poultry, eggs, reduced or low-fat dairy products, regular or high-fat dairy products, legumes, nuts, refined grain products and whole grains products. Total animal protein and total vegetal protein (in grams) were also available from the database by calculating the sum of each food sources and mixed dishes. Nutritional information from foods missing in the database was derived from nutritional food labels and entered manually. 

### 2.3. Anthropometric Measurements

Participants were asked to wear light indoor clothes on the day of their appointment. All measurements were made by a trained research assistant. Weight and height were measured using a beam scale with rod graduated in centimetres (Detecto, Webb City, MO USA). Weight was measured to the nearest 0.1 kg and height was measured to the nearest 0.5 cm. Body mass index (BMI) was computed as weight in kilograms divided by height in meters squared (kg/m^2^). OW was defined as having BMI over 25 kg/m^2^ while individuals with a BMI below 25 kg/m^2^ were defined as having a NW. Waist (WC) and hip circumferences were measured according to the procedures recommended by the Airlie Conference [38]. For the measure of systolic (SBP) and diastolic blood pressure (DBP), participants were asked to sit straight with arms and legs uncrossed. The measures were taken after a 5-minute rest. 

### 2.4. Biochemical Parameters

Blood samples were collected from an antecubital vein into vacutainer tubes containing EDTA after a 12-h overnight fast. Blood samples were immediately centrifuged. Total cholesterol (total-C) and triglyceride (TG) concentrations were determined from plasma and lipoprotein fractions using the Olympus AU400e system (Olympus America Inc., Melville, NY, USA). A precipitation of low-density lipoprotein cholesterol (LDL-C) fraction in the infranatant with heparin-manganese chloride was used to obtain the high-density lipoprotein cholesterol (HDL-C) fraction. LDL-C concentrations were estimated using the Friedewald’s equation [39]. Radioimmunoassay with polyethylene glycol separation was used to measure fasting insulin. Fasting glucose concentrations were enzymatically measured. Homeostasis model assessment of IR (HOMA-IR) was obtained using (fasting glucose × fasting insulin)/22.5. MS was defined as having three or more of the following risk factors: WC >88 cm for women and 102 cm for men, fasting plasma TG ≥1.7 mmol/L, HDL-C levels ≤1.29 mmol/L for women and 1.03 mmol/L for men, glucose levels ≥5.6 mmol/L and resting SBP/DBP ≥130/85 mmHg. Participants taking medication for lipidemia, diabetes or hypertension control were considered as having abnormal values for their respective parameters. 

### 2.5. Metabolite Profiling

As previously described [35], the Absolute ID p180 Kit (Biocrates Life Sciences AG, Innsbruck, Australia) for mass spectrometry was used for the metabolic profiling measurements for two-hundred participants. Ninety-five metabolites were quantified. They include: 67 Glycerophospholipids (GPs), 12 AC, 10 Sphingolipids (SGs) and 6 AAs. For GPs, ACs and SGs x:y notation was used, x denoting the number of carbons in the side chain and y the number of double bonds. All metabolite concentrations are presented in μM. A metabolite would have been excluded if more than half of the values obtained were below the limit of detection or with standard out of range.

### 2.6. Statistical Analyses

Variables not normally distributed were transformed using log_10_ (TG, HDL-C, insulin, animal protein, processed meat, eggs, low-fat dairy), square root (legumes) or inverse transformation (nuts). Organ meat intakes were still not normally distributed after transformation and were then used as a categorical variable (eater or non-eater of organ meat). BCAA dietary intakes or plasma levels were defined as the sum of valine, leucine and isoleucine respectively calculated in FFQ or following plasma metabolite profiling. The General Linear Model (GLM) procedure with the type-III sum of squares was used to assess the association between plasma BCAA levels and age, sex, BMI, WC, BCAA intakes, energy from proteins and total energy intakes. Different models were computed to further assess the associations between plasma BCAA levels, vegetal protein and animal protein and their constituents, as well as to take into account adjustments for total daily energy intakes, age and sex. Associations between different protein sources, as well as BCAA intakes and plasma BCAA levels, were tested with and without adjustments for confounding factors. The associations between ACs levels and plasma BCAA levels were finally assessed. The same models were tested when subdividing study participants based on OW status and the absence/presence of MS (MS−/MS+). Four groups were consequently created: NW/MS−, NW/MS+, OW/MS− and OW/MS+. The NW/MS+ group was excluded from dietary, BCAAs and ACs analyses since this group was composed of only one woman and one man. The GLM procedure was also used to compare mean intakes between groups. All data analyses were performed using SAS statistical software University edition (SAS Institute Inc, Cary, NC, USA). A *p*-value < 0.05 was considered as statistically significant.

## 3. Results

### 3.1. Study Population

Characteristics of study participants are presented in Table 1. Mean values for the four groups are presented. Mean age of participants was 34.2 years and 49.7% of them were women. There were significant differences between groups for all anthropometric and metabolic parameters except for men/women proportions and LDL-C levels. OW individuals were older and had higher BMI, WC, total-C, TG, insulin, SBP and DBP, as well as lower HDL-C than NW/MS− subjects.

### 3.2. Dietary Intakes

As shown in Table 2, mean daily protein intakes were 104.2 g, which represents 16.8% of total daily energy intakes. Proteins were mainly provided by animal-based foods, with a mean intake of 70.5g versus 32.0g from plant-based sources. There were some differences between groups for total energy (*p* = 0.0172), total carbohydrates (*p* = 0.0446), % of kcal from carbohydrates (*p* = 0.0340), total protein (*p* = 0.0303), animal protein (*p* = 0.0086), BCAA intakes (0.0310), total fat (*p* = 0.0102), total SFA (*p* = 0.0173), total monounsaturated fatty acids (MUFA) (*p* = 0.0131) and total polyunsaturated fatty acids (PUFA) (*p* = 0.0347) intakes. These differences were no longer significant after adjustments for age, sex and energy intake except for significantly greater animal protein intakes in both OW groups (*p* = 0.0388). Table 3 presents mean intakes, expressed in portions/day, of the food subgroups providing most dietary proteins. OW individuals either MS- or MS+ consumed less fish (*p* = 0.0106) and more eggs (*p* = 0.0166) than NW/MS− individuals, but the difference in eggs consumption did not remain significant after adjustments for age, sex and total energy intake. OW/MS+ also consumed more red meat (*p* = 0.0027) and this was still observed, but only as a trend, after adjustments for age, sex and total energy intake (*p* = 0.0899). A detailed list of foods included in each category is provided in Supplementary Table S1.

### 3.3. Plasma BCAAs and Protein Intakes

**Figure 1:**
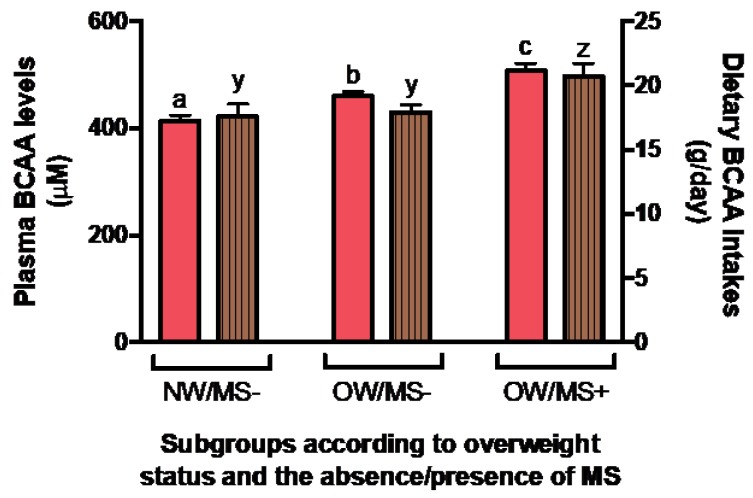
Mean plasma and dietary BCAA levels between subgroups of NW/OW individuals with or without MS. Plasma BCAA levels are shown in plain salmon; BCAA intakes calculated from food frequency questionnaire are shown in lined brown. Whiskers represent standard error. Results who do not share the same letter (^a,b,c^ for plasma BCAA levels and ^y,z^ for dietary BCAA intakes) are significantly different (*p* < 0.05) from each other. Values presented are unadjusted.

The associations between total protein intake, total energy, BCAA intakes, age, sex and BMI with plasma BCAA levels have also been investigated. Sex and BMI or WC contributed significantly to the variance of plasma BCAA levels (*p* < 0.0001 for both), while total protein, total energy and BCAA intakes and age did not. We also investigated plasma BCAA levels according to obesity and MS status. As shown in Figure 1, there was an increase of plasma BCAA levels with obesity and the presence of MS (*p* < 0.0001) that remained significant after adjustments for age, sex and energy intake (*p* < 0.0001). A concomitant increase in BCAA intakes was also seen (*p* = 0.0310), with OW/MS+ consuming more BCAAs than the other groups (Figure 1). However, the difference in BCAA intakes was no longer significant after adjustments for confounding factors, including age, sex and energy intake (*p* = 0.3789). 

The association of each of the 12 principal protein sources (red meat, processed meat, fish, poultry, eggs, legumes, nuts, high and low-fat dairy and whole and refined grain products) with plasma BCAA levels was also tested in a GLM model. Considering all 197 study participants, the only trend observed was with red meat (*p* = 0.0575). This trend was lost after adjustments for age, sex, BMI and total energy intake (data not shown).

When looking at food correlates of plasma BCAA levels in groups defined on the basis of OW and MS status (Table 4), refined and whole grain products were positively associated with plasma BCAA levels (β = 16.07, *p* = 0.0338 and β = 13.04, *p* = 0.0279, respectively) in NW/MS− subjects. Their respective contribution to the variance of plasma BCAA levels was of 5.55% and 5.97%, respectively. None of the food group was significantly associated with plasma BCAA levels in OW/MS+ subjects, but a positive trend was seen for red meat (β = 15.40, *p* = 0.0713). After adjustments for age, sex and energy intake, a negative association between red meat consumption and plasma BCAA levels (β = −26.15, *p* = 0.0013) was found in NW/MS, explaining 9.64% of its variance. When looking at men and women separately, this negative association was only found in NW/MS− men (β = −49.75.16, *p* = 0.0039) and not in NW/MS− women (β = −11.81, *p* = 0.4715). As for OW/MS+ individuals, red meat showed a trend toward a positive relationship (β = 16.16, *p* = 0.0548), and a negative association was observed with eggs (β = −310.14, *p* = 0.0272). 

Finally, in a model testing the association between total animal and vegetal protein intakes and plasma BCAA levels, only animal protein intake was associated with plasma BCAA levels (*p* = 0.0002) with a weak contribution of 6.89% to the variance of the trait (not shown). After adjustments for age, sex and energy, the positive association between animal protein intakes and plasma BCAA levels remained significant (R^2^ = 0.0193, *p* = 0.0297, not shown). When analysed by sex, this association was significant in women (β = 177.16, *p* = 0.0164), but not in men (β = 98.12, *p* = 0.2908). In subgroups, there was a positive association between total animal protein intakes and plasma BCAA levels for NW/MS− (R^2^ = 0.0675, *p* = 0.0292), as well as a trend toward relationship for OW/MS+ (R^2^ = 0.0664, *p* = 0.0786) individuals. After adjustments for age, sex and energy intake, the positive association between animal protein intakes and plasma BCAA levels was significant in OW/MS+ (R^2^ = 0.0422, *p* = 0.0422), but was lost in NW/MS− individuals (not shown). 

As animal protein is the main nutritional correlate of plasma BCAA levels, we further tested the association with its constituents, thus including red meat, processed and organ meats, fish, poultry, eggs and low and high fat dairy in the model. Again, red meat was the single constituent significantly and positively associated with plasma BCAA levels (*p* = 0.0388) while a positive trend was also observed with poultry (*p* = 0.0801). These associations (with respective R^2^ of 0.0209 and 0.0150) were no longer significant after adjustments for age, sex and total energy intake (data not shown). 

### 3.4. Acylcarnitines 

**Figure 2:**
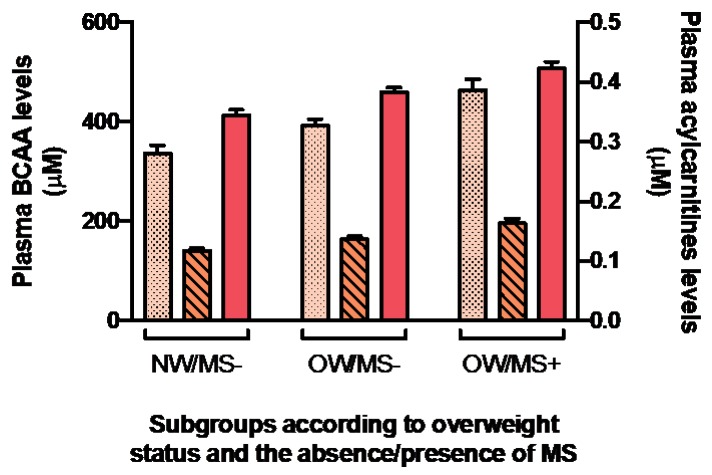
Mean plasma concentrations of C3 and C5 ACs in relation with mean plasma BCAA levels between subgroups of NW/OW individual with or without MS. Plasma BCAA levels are shown in salmon; C3 ACs are shown in dotted light orange; C5 ACs are shown in striped medium orange. Whiskers represent standard error. Values presented are unadjusted. All mean values are significantly different (*p* < 0.05) between groups.

As shown in Figure 2, plasma concentrations of both C3 and C5 ACs increased in parallel to plasma BCAA levels according to OW status and MS even after adjustments for age, sex and total energy intake (*p* < 0.001) (not shown). The associations between plasma BCAA and plasma C3 and C5 AC levels were also tested. With or without adjustments for age, sex and total energy intake, associations between plasma BCAAs and C3 and C5 ACs were significant in the whole cohort (*p* < 0.0001 for all), and in subgroups based on obesity and MS presence (*p* < 0.002 for all) (Table 5). After further adjustments for dietary BCAAs, red meat and total protein intakes, only the association between plasma BCAAs and C5 ACs in OW/MS+ was lost. The contribution of C3 and C5 ACs to the variance was weak to moderate, with R^2^ values ranging from 0.0466 to 0.3817. 

## 4. Discussion

It is not yet fully established if elevated plasma BCAA and AC levels are a cause or a consequence of IR, and if protein intakes exert an influence on their plasma concentrations. To our knowledge, we are the first to investigate the association between the individual food groups contributing the most to daily protein intakes, fasting AC and BCAA levels in normal weight and overweight/obese individuals with or without metabolic perturbations.

As expected, plasma BCAA levels were significantly greater in OW with or without MS than in NW individuals, which is consistent with the literature [22,40,41,42,43]. Worth of mention, plasma BCAAs appeared to be affected by sex, as previously reported in some studies [40,41,42]. However, differences in BCAA intakes between groups were not present following adjustments for age, sex and energy intake. This was expected since total protein intakes and % of energy from protein were also not different between groups after adjustments. Still, the protein’s origin deserves further attention. Considering all principal protein sources, red meat seems to be driving the positive association of total animal protein intakes with plasma BCAAs since it was the only food group presenting a trend towards significance for an association with plasma BCAAs. Associations with red meat were lost in adjusted models on the whole sample, but animal protein intakes remained significant. This reflects very well the intakes of our participants: OW/MS+ consumed significantly more animal protein than NW/MS− and OW/MS− individuals in non-adjusted and adjusted models, whereas red meat intakes were no longer different between groups after adjustments for age, sex and energy. Considering these observations, plasma BCAA levels potentially reflect the consumption of animal protein/red meat. Greater red meat intakes would have been necessary to see an effect of this food on already metabolically deteriorated individuals. These findings are concordant with recent papers reporting BCAAs consumption—correlating with animal protein and/or meat intakes—associated or correlated with plasma BCAA levels [8,17]. Still, because animal protein and red meat only explain a small portion of plasma BCAAs variance, dietary intakes might not be the main variable affecting plasma BCAA levels.

Testing the associations within the three subgroups of subjects revealed substantial differences. In OW/MS+ individuals, the association between plasma BCAAs and animal protein presented a trend toward significance that became significant after adjustments. In the model including all 12 principal protein sources, the tendency observed for red meat persisted after adjustments, but reached significance when considering animal protein sources only (not shown). Similarly to what we observed in the whole sample, red meat was the main animal protein source associated with plasma BCAA levels in metabolically disturbed individuals although the small magnitude of the relationship is probably an indicator that there are other important metabolic factors implicated in plasma BCAAs elevation in this group. 

As for OW/MS−, we surprisingly did not find any association of protein intakes with plasma BCAA levels. Their intakes were more similar to NW than to OW/MS+ individuals regarding macronutrients, but were no longer significantly different after adjustments for age, sex and energy intake. Yet, compared to their MS+ counterparts, they consumed less red meat and less animal protein overall. The hypothesis that elevated plasma BCAAs is consequent to IR could explain why their levels are significantly higher than NW/MS− subjects, assuming that they are at a greater risk of developing IR. At this point, we cannot rule out the possibility that the different dietary habits of OW/MS− exert some kind of protection against elevated plasma BCAA levels. Unfortunately, the study design of the INFOGENE study does not allow the verification of this hypothesis.

What has been observed in NW/MS− individuals is quite different. All models presented different associations. In the unadjusted ones, grain products were the main protein sources positively associated with plasma BCAA levels while total animal protein, but not vegetal protein intakes, was also positively associated. This unexpected observation could rather be an indicator of a dietary pattern rich in grain products, as well as in animal protein, explaining their associations with plasma BCAAs in different models. Even more intriguing, after adjustments for confounders, red meat was negatively associated with plasma BCAA levels. Sex appeared to be a moderator of this association (*p* < 0.0001). BCAAs might be more strongly affected by sex (and other metabolic factors) than by protein sources intakes in healthy individuals. This could explain why we found very different associations depending on the model used and would corroborate with the small magnitude of the associations found with the 12 principal protein food sources intakes. Of note, a decreased predictive effect of the habitual diet on serum metabolites after sex and age adjustments was previously reported by Floegel et al [44]. 

Plasma AC levels were another important aspect of the present study. It appears that the association between plasma C3 and C5 ACs and plasma BCAA levels were influenced by sex but not so much by other confounders, such as age, energy intakes, animal protein, red meat or BCAA intakes, depending on the model used. Further adjustments for variables related to dietary protein intakes did not change the associations between C3 or C5 ACs and plasma BCAA levels except for the loss of the association for C5 ACs in OW/MS+. In that model, neither animal protein, red meat or BCAA intakes were significant correlates to the variance. Thus, we cannot confirm if these variables modulate the association between C5 ACs and plasma BCAA levels or if we lost the association because of a lack of statistical power, but their contribution to the variance appears to be weak. C3 and C5 ACs might be differentially associated with plasma BCAA, but literature does not report on their individual effect [25,44]. We found C3 and C5 ACs to be positively associated with plasma BCAAs as reported in a comparable study where higher levels of short-chain ACs were observed in diabetic patients compared to lean or obese individuals [45]. Since this association persisted with adjustments for confounders and animal protein, BCAA and red meat intakes in our models, there appears to be a relative independence of C3 and C5 ACs from the diet. Consequently, we propose that C3 and C5 ACs more likely represents the degree of plasma BCAAs elevation than meat consumption. For these reasons, using these short-chain ACs as a biomarker of meat consumption should be done with caution and with consideration of other metabolic markers. It would be interesting to further investigate these association in fasting and non-fasting individuals since C3 and C5 levels could be lower in fasted individuals [46].

Taken all together, these findings are in line with a study realized in a cohort of Asian Indians living in the US and at risk of CVD [47]. The investigators reported a positive association between a Western/non-vegetarian dietary pattern, characterized by higher intakes of red meat, poultry, fish, eggs and vegetables, and a metabolite signature rich in BCAAs, as well as in aromatic AAs and short-chain ACs. Participants scoring higher for that metabolite signature were also more insulin resistant, had higher fasting and 2-h insulin concentrations and had lower adiponectin levels and insulin sensitivity. Similarly, we found a metabolic signature, including BCAA leucine, C3 and C5 ACs that was associated with a Western dietary pattern in French Canadians [24]. Red meat also appeared to be an important positive loading factor for T2D risk [44]. As for our healthy volunteers, recent work found meats, sausages, meat products and eggs to be included in dietary patterns explaining variation among plasma BCAA levels in a healthy population [17]. These foods were highlighted in at least one of our models. 

The mechanisms underlying the association between BCAA intakes and IR development are unclear for now and association studies reported opposite results [4,28]. The largest European prospective study found total and mostly animal protein intakes to be associated with an elevated T2D risk [48]. As observed in the present study, and in similar studies [17,18,19,20], plant-protein intakes were not inversely associated with T2D risk. Interestingly, Okekunle et al., found that a pattern rich in meat was associated with T2D risk, even if rice and wheaten foods were the main correlates of BCAAs in their study cohort [49]. These findings, alongside the higher intakes of animal protein observed in OW/MS+ individuals herein, reiterate the importance of considering the composition of the foods BCAAs are coming from. In fact, red meat does have high heme-iron content which was suggested to be associated with T2D [50,51,52,53,54]. Other nutrients in meats may also have an impact on IR development, including the pro-oxidant and pro-inflammatory advanced glycation end products formed by the reaction of carbonyl groups of reducing sugars with the amine groups of proteins, lipids or nucleotides [55,56]. We also cannot exclude the role of the gut microbiota (GMB) in BCAAs metabolism. Accordingly, intestinal bacteria impact AAs absorption that are used for growth or the synthesis of metabolic compounds, such as short-chain fatty acids (SCFA), including branched-chain fatty acids (BCFA), BCAA being involved in the later [57]. Finally, animal protein could have a different biodisponibility or kinetics that could predispose to IR. A small study reported that vegan, who consumed less BCAAs than omnivores at baseline, had a significant decrease in insulin sensitivity after three months of BCAA supplementation [26]. Another study found a switch to fish and plant-based protein versus meat lowered plasma BCAAs in a small sample of mainly overweight individuals [58]. Mechanisms of actions need to be the subject of future studies. 

All things considered, results of the present study and a literature review lead us to two different hypotheses: First, that the protein quality has an impact on plasma BCAA levels and second, that elevated BCAAs are the consequence of a disturbed metabolism in individuals having suboptimal dietary habits. In both cases, ACs seems more likely to reflect BCAA concentrations in the plasma. If BCAA levels induce IR, the most plausible hypothesis involves the mammalian target of rapamycin (mTOR) [59,60]. BCAAs and especially leucine can activate mTORC1 through an alternative pathway ending with insulin receptor degradation. Insulin-binding to its receptor being therefore compromised, a state of insulin resistance would occur [59,60,61,62,63,64,65,66]. BCAAs could also increase the activity of p65 subunit of nuclear transcription factor Kb (NF-kβ), a pro-inflammatory pathway that could accelerate IR progression [67]. Regardless of their potential, these mechanisms might not explain the rise of plasma BCAA levels observed in our sample, granted that protein intakes and therefore amino acid pool, were not greater in OW/MS− compared to NW/MS− group. If BCAAs elevation is rather the consequence of a disturbed metabolism, it has been proposed that high fat diets, obesity, IR or insulin levels could lead to a defect in BCKD activity and expression in the liver [11]. Circulating BCAAs are transaminated into branched-chain keto acids (BCKA) by the branched-chain amino acid transferase, a reversible step, before being further oxidized by BCKD to serve as substrates in the Krebs cycle [2,11,68]. Having dysfunctional BCKD would therefore lead to BCKA and or BCAAs accumulation. BCKA being the precursors of C3 and C5 ACs, this could explain ACs elevation in conjunction with IR [2]. 

The present study has some limitations. As mentioned earlier, elevated BCAAs is an early predictor of IR. Individuals classified in the MS− group could be insulin resistant but without enough metabolic impairments to make it into the MS+ group and therefore dilute IR impact on our results. As previously hypothesized by Isanejad et al., the response to dietary protein might depend on metabolic health, as well as on one’s degree of IR [19]. Classifying our population according to IR only could help to isolate the impact of protein intakes on BCAAs at different stages of diabetes development (healthy, insulin resistant and diabetic). Also, we considered the medication for diabetes, dyslipidemia or hypertension to define our subgroups. But taking medications artificially improve metabolic parameters, which could therefore ameliorate BCAAs metabolism and affect the associations observed. Our sample was also too small for further investigation of the effect of sex on plasma BCAA levels by subgroups based on BMI and MS presence. Finally, because we used data from a cross-sectional study, we were not able to investigate in a prospective way the associations between plasma BCAA levels and changes in consumption of plant or animal protein nor time variations in ACs. It is important to note that correction for multiple testing was not applied to data. Because of the exploratory design of this study, having applied too restrictive correction could have masked potentially interesting associations in this relatively small sample of subjects. Notwithstanding, this study has some strengths. Our decision to compare healthy and metabolically deteriorated individuals was based on the fact that plasma BCAA levels are influenced by other components of the metabolic syndrome than glycemia [2]. As such, the present work compares healthy and metabolically deteriorated individuals within a single sample allowing comparisons between groups and generalization of the results. To our knowledge, it is also the first study testing the association of BCAAs with diet between subgroups of subjects with cardiometabolic perturbations as opposed to only IR or CVD related risk factors. All measurements were standardized, including the dietary questionnaire that has been validated for our population. Finally, the detailed FFQ allowed us to explore the associations with specific subgroups of animal-derived protein sources in parallel with ACs and BCAAs, which is unique to the present study. 

## 5. Conclusions

In summary, we found a constant tendency toward significance between plasma BCAAs and animal protein or red meat intakes in OW/MS+ individuals. In NW/MS− individuals, diverse associations were observed between models. That being said, it is likely that BCAA or animal protein/meat intakes are not the main or the sole correlate to their elevation in plasma prior to IR considering their weak contribution to the variance. Plasma ACs concentrations were also found to be associated with plasma BCAA levels. Our study cannot explain mechanisms by which plasma BCAA levels and ACs are elevated in OW/MS+ individuals, but the impact of meat, its BCAA content, the presence of other compounds in its matrix and its GMB metabolites should be further investigated. 

## Figures and Tables

**Table 1:** Characteristics of participants.

Characteristics	Total Subjects (*n* = 199)	NW/MS− (*n* = 65)	NW/MS+ (*n* = 2)	OW/MS− (*n* = 84)	OW/MS+ (*n* = 48)	*p*-Value
Women (%)	49.7	58.5	50.0	51.1	35.4	0.1130
Age (years)	34.2 ± 10.2	28.9 ± 7.4 ^a^	39.1 ± 17.8 ^a^	35.7 ± 10.4 ^b^	38.4 ± 10.1 ^b,c^	**<0.0001**
BMI (kg/m^2^)	29.0 ± 6.2	22.2 ± 1.8 ^a^	24.5 ± 0.66 ^a^	31.4 ± 4.2 ^b^	34.3 ± 4.8 ^c^	**<0.0001**
WC (cm)	92.7 ± 16.5	74.7 ± 5.7 ^a^	83.1 ± 0.64 ^a^	97.7 ± 10.7 ^b^	109.0 ± 11.8 ^c^	**<0.0001**
Total-C (mmol)	4.49 ± 1.00	4.13 ± 0.67 ^a^	3.58 ± 0.76 ^a,b^	4.57 ± 1.0 ^b^	4.88 ± 1.19 ^b^	**0.0003**
TG (mmol)	1.29 ± 0.91	0.77 ± 0.31 ^a^	0.88 ± 0.03 ^a,b^	1.15 ± 0.56 ^b,c^	2.23 ± 1.22 ^d^	**<0.0001** *
HDL-C (mmol)	1.33 ± 0.41	1.60 ± 0.45^c^	1.07 ± 0.21 ^a,b^	1.32 ± 0.30 ^b^	0.99 ± 0.24 ^a^	**<0.0001** *
LDL-C (mmol)	2.76 ± 0.94	2.52 ± 0.70	2.51 ± 0.55	2.81 ± 0.91	3.00 ± 1.21	0.0545
Fasting glycemia (mmol/L)	5.65 ± 0.74	5.68 ± 0.74 ^a^	6.90 ± 1.13 ^b^	5.43 ± 0.52 ^c^	5.94 ± 1.92 ^a,b^	**0.0001**
Insulin (pM)	85.1 ± 60.6	48.7 ± 17.1 ^a^	68.5 ± 21.9 ^a,b,c^	86.1 ± 56.3 ^b^	134.5 ± 72.2 ^c^	**<0.0001** *
SBP (mmHg)	121.3 ± 11.1	115.9 ± 9.8 ^a^	130.5 ± 4.9 ^b,c,d^	119.9 ± 8.9 ^c^	130.5 ± 10.6 ^d^	**<0.0001**
DBP (mmHg)	77.9 ± 9.5	74.0 ± 9.9 ^a^	74.0 ± 5.7 ^a,b,c^	77.7 ± 7.7 ^b^	83.6 ± 9.4 ^c^	**<0.0001**
BCAAs (µM)	455.6 ± 92.3	413.8 ± 83.5 ^a^	378.8 ± 83.3 ^a,b^	460.0 ± 83.2 ^a,b^	507.7 ± 92.3 ^b^	**<0.0001**
C3 ACs (µM)	0.325 ± 0.115	0.281 ± 0.104 ^a^	0.181 ± 0.011 ^a,b^	0.327 ± 0.100 ^b^	0.387 ± 0.125 ^c^	**<0.0001**
C5 ACs (µM)	0.137 ±0.048	0.118±0.036 ^a^	0.094± 0.005 ^a,b^	0.137 ± 0.045 ^b^	0.163 ± 0.056 ^c^	**<0.0001**

Values are means ± SD. Model *p*-values of comparisons between NW/MS−, NW/MS+, OW/MS− and MW/MS+ are shown. Between-groups comparisons were made using the LS means procedure. Results who do not share the same letter (^a,b,c,d^) are significantly different (*p* < 0.05) from each other. * indicates that the *p*-value was obtained with the transformed variables. Significant values (*p* < 0.05) are presented in bold. Abbreviations, NW, normal weight; OW, overweight; MS, metabolic syndrome; BMI, body mass index; WC, waist circumference; Total-C, total cholesterol; TG, triglycerides; HDL-C, high-density lipoproteins; LDL-C, low-density lipoproteins; SBP, systolic blood pressure; DBP, diastolic blood pressure; BCAAs, branched-chain amino-acids; ACs, acylcarnitines; SD, standard deviation.

**Table 2:** Daily dietary energy and macronutrients intakes of participants.

Nutrients	Total Subjects (*n* = 197)	NW/MS− (*n* = 65)	OW/MS− (*n* = 84)	OW/MS+ (*n* = 48)	*p*-Value ^1^	*p*-Value ^2^
Total energy (kcal)	2474 ± 790	2412 ± 853 ^a^	2364 ± 695 ^a^	2754 ± 809 ^b^	**0.0172**	
Total carbohydrates (g)	297.5 ± 92.5	298.8 ± 95.7 ^a,c^	281.7 ± 87.0 ^a,b^	323.3 ± 93.5 ^c^	**0.0446**	0.5447
Carbohydrates (%kcal)	46.7 ± 6.0	48.23 ± 6.54 ^a^	45.9 ± 5.3 ^b^	45.8 ± 6.1 ^b^	**0.0340**	0.2229
Total dietary fiber (g)	23.5 ± 7.9	23.6 ± 8.7	22.8 ± 7.3	24.6 ± 7.7	0.4495	0.7690
Soluble dietary fibers (g)	7.8 ± 2.5	7.6 ± 2.6	7.5 ± 2.3	8.5 ± 2.7	0.0745	0.7736
Insoluble dietary fibers (g)	15.5 ± 5.5	15.7 ± 6.2	15.2 ± 5.2	15.9 ± 5.1	0.7463	0.5677
Total protein (g)	104.2 ± 37.1	99.9 ± 43.2 ^a^	100.4 ± 29.9 ^a^	116.4 ± 37.7 ^b^	**0.0303**	0.6209
Protein (%kcal)	16.8 ± 2.4	16.4 ± 2.6	17.1 ± 2.0	17.0 ± 2.5	0.2580	0.3762
Vegetal protein (g)	32.0 ± 11.8	31.9 ± 12.5	31.0 ± 11.6	34.1 ± 11.1	0.3459	0.6093
Animal protein (g)	70.5 ± 30.3	66.2 ± 35.9 ^a^	68.0 ± 23.7 ^a^	80.7 ± 30.9 ^b^	**0.0086** *	0.0388 *
BCAA intakes (g)	18.5 ± 6.8	17.6 ± 7.8 ^a^	17.9 ± 5.5 ^a^	20.7 ± 7.0 ^b^	**0.0310**	0.3789
Total fat (g)	93.4 ± 37.3	88.4 ± 39.9 ^a^	89.2 ± 30.6 ^a^	107.4 ± 41.3 ^b^	**0.0102**	0.8410
Fat (%kcal)	33.6 ± 5.3	32.4 ± 5.7	33.8 ± 4.7	34.7 ± 5.6	0.0649	0.5095
Total SFA (g)	32.6 ± 14.7	30.6 ± 15.9 ^a^	31.1 ± 12.3 ^a^	37.8 ± 16.0 ^b^	**0.0173**	0.8734
SFA (%kcal)	11.6 ± 2.6	11.1 ± 2.7	11.8 ± 2.6	12.1 ± 2.6	0.0902	0.4312
Total MUFA (g)	38.3 ± 15.7	36.3 ± 16.7 ^a^	36.5 ± 12.5 ^a^	44.0 ± 18.1 ^b^	**0.0131**	0.9269
MUFA (%kcal)	13.8 ± 2.6	13.3 ± 2.9	13.9 ± 2.2	14.2 ± 2.8	0.2203	0.7753
Total PUFA (g)	15.2 ± 6.2	14.5 ± 6.3 ^a^	14.7 ± 5.6 ^a^	17.3 ± 6.9 ^b^	**0.0347**	0.9148
PUFA (%kcal)	5.5 ± 1.4	5.4 ± 1.3	5.6 ± 1.3	5.6 ± 1.5	0.6765	0.9631
Total alcohol (g)	10.5 ± 12.1	9.8 ± 9.0	11.1 ± 13.0	10.5 ± 14.0	0.2968 *	0.1406 *
Alcohol (%kcal)	3.0 ± 2.9	2.9 ± 2.5	3.2 ± 3.2	2.5 ± 2.9	0.4770	0.3919

Values are means ± SD. %kcal from carbohydrates was calculated by difference. Model *p*-values of comparisons between NW/MS−, OW/MS− and MW/MS+ are shown ^1^unadjusted and ^2^adjusted for age, sex and total energy intakes. Between-groups comparisons were made using the LS means procedure. Results who do not share the same letter (^a,b,c^) are significantly different (*p* < 0.05) from each other. * indicates that the *p*-value was obtained with the transformed variables. Significant values (*p* < 0.05) are presented in bold. Abbreviations: NW, normal weight; OW, overweight; MS, metabolic syndrome; BCAA, branched-chain amino-acids; SFA, saturated fatty acids; MUFA, monounsaturated fatty acids; PUFA, polyunsaturated fatty acids.

**Table 3:** Mean intakes (standard portions/day) of the principal food groups contributing to protein intakes

Food Groups	Total Subjects (*n* = 197)	NW/MS− (*n* = 65)	OW/MS− (*n* = 84)	OW/MS+ (*n* = 48)	*p*-Value ^1^	*p*-Value ^2^
Red meat	2.25 ± 1.78	1.93 ± 1.95 ^a^	2.07 ± 1.40 ^a^	3.01 ± 1.94 ^b^	**0.0027**	0.0899
Processed meat	0.85 ± 0.95	0.78 ± 0.83	0.72 ± 0.60	1.16 ± 1.44	0.0660 *	0.4325 *
Organ meat	0.02 ± 0.09	0.01 ± 0.04	0.03 ± 0.10	0.04 ± 0.11	0.1009 *	0.2641 *
Fish	1.21 ± 1.19	1.56 ± 1.56 ^b^	1.11 ± 0.97 ^a^	0.92 ± 0.81 ^a^	0.0106	**0.0119**
Poultry	1.17 ± 0.91	1.13 ± 0.94	1.23 ± 0.89	1.12 ± 0.90	0.7332	0.1337
Eggs	0.36 ± 0.30	0.28 ± 0.23 ^a^	0.38 ± 0.29 ^b^	0.43 ± 0.37 ^b^	**0.0166**	0.1431
Low fat dairy	1.60 ± 1.34	1.42 ± 1.08	1.59 ± 1.24	1.86 ± 1.77	0.5081 *	0.5713 *
High fat dairy	1.80 ± 1.29	1.73 ± 1.27	1.77 ± 1.17	1.95 ± 1.51	0.6389	0.7883
Legumes	0.28 ± 0.49	0.28 ± 0.41	0.31 ± 0.62	0.21 ± 0.32	0.4132 *	0.2473 *
Nuts	0.97 ± 2.99	0.75 ± 0.81	0.80 ± 1.00	1.58 ± 5.84	0.9254 *	0.9691 *
Refined grain products	2.78 ± 1.95	2.72 ± 1.65	2.54 ± 1.83	3.28 ± 2.41	0.1028	0.7890
Whole grain products	2.43 ± 1.85	2.64 ± 2.13	2.34 ± 1.63	2.29 ± 1.82	0.5212	0.1007

Values are means ± SD. ^1^ *p*-value unadjusted, ^2^ *p*-value adjusted for age, sex and total energy intakes. Results who do not share the same letter (^a,b^) are significantly different (*p* < 0.05) from each other. * indicates that the *p*-value was obtained with the transformed variables. Significant values (*p* < 0.05) are presented in bold. Abbreviations: NW, normal weight; OW, overweight; MS, metabolic syndrome.

**Table 4:** Associations between all 12 principal protein sources and plasma BCAA levels in subgroups based on OW and MS status without and with adjustments for confounders.

Group	NW/MS− (*n* = 65)				OW/MS− (*n* = 84)				OW/MS+ (*n* = 48)			
Model	Unadjusted		Adjusted ^2^		Unadjusted		Adjusted ^2^		Unadjusted		Adjusted ^2^	
Parameters	R^2^	*p*-value	R^2^	*p*-value	R^2^	*p*-value	R^2^	*p*-value	R^2^	*p*-value	R^2^	*p*-value
Red meat	0.0087	0.3910	0.0964	**0.0013**	0.0113	0.3408	0.0072	0.4330	0.0771	0.0713	0.0602	0.0548
Processed meat *	0.0088	0.3892	0.0047	0.4571	0.0004	0.8625	0.0005	0.8336	0.0025	0.7380	0.0002	0.9061
Organ meat *	0.0007	0.8112	0.0006	0.7977	0.0028	0.6313	0.0002	0.8871	0.0293	0.2591	0.0449	0.0948
Fish	0.0258	0.1429	0.0306	0.0607	0.0105	0.3589	0.0037	0.5756	0.0151	0.4160	0.0149	0.3288
Poultry	0.0197	0.1999	0.0219	0.1108	0.0046	0.5410	0.0004	0.8479	0.0397	0.1902	0.0429	0.1022
Eggs *	0.0203	0.1936	0.0214	0.1153	0.0076	0.4324	0.0074	0.4275	0.0578	0.1161	0.0812	**0.0272**
Legumes *	0.0140	0.2787	0.0003	0.8594	0.0072	0.4453	0.0066	0.4543	0.0047	0.6496	0.0424	0.1039
Nuts *	0.0164	0.2418	0.0065	0.3820	0.0045	0.5485	0.0032	0.6039	0.0049	0.6432	0.0001	0.9438
Hf dairy	0.0071	0.4395	0.0058	0.4077	0.0296	0.1248	0.0344	0.0904	0.0105	0.4973	0.0099	0.4244
Lf dairy *	0.0010	0.7721	0.0006	0.7817	0.0013	0.7469	0.0088	0.3890	0.0001	0.9434	0.0054	0.5539
Refined gp	0.0555	**0.0338**	0.0016	0.6596	0.0146	0.2785	0.0000	0.9857	0.0207	0.3415	0.0108	0.4047
Whole gp	0.0597	**0.0279**	0.0002	0.8773	0.0161	0.2552	0.0020	0.6791	0.0250	0.2963	0.0053	0.5576

Protein sources were analysed in portions/day. ^2^ The model is adjusted for age, sex and total energy intakes. * indicates that the values was obtained with the transformed variables. Significant values (*p* < 0.05) are presented in bold. Abbreviations: NW, normal weight; OW, overweight; MS, metabolic syndrome; Hf dairy, high fat dairy; Lf dairy, Low fat dairy; Refined gp, refined grain products; Whole gp, whole grain products.

**Table 5:** Associations between plasma BCAA and C3 and C5 ACs plasma levels in the whole cohort and by subgroups based on OW and MS status without and with adjustments for confounders. All lines were computed individually.

		Total cohort (*n* = 197)		NW/MS− (*n* = 65)		OW/MS− (*n* = 84)		OW/MS+ (*n* = 48)	
Parameters	Model	R^2^	*p*-value	R^2^	*p*-value	R^2^	*p*-value	R^2^	*p*-value
C3 ACs	Unadjusted	0.3804	**<0.0001**	0.3529	**<0.0001**	0.3069	**<0.0001**	0.2812	**0.0001**
	Adjusted ^2^	0.2080	**<0.0001**	0.0909	**0.0020**	0.2356	**<0.0001**	0.2181	**0.0002**
	Adjusted ^3^	0.1932	**<0.0001**	0.0775	**0.0047**	0.2140	**<0.0001**	0.1488	**0.0019**
C5 ACs	Unadjusted	0.3817	**<0.0001**	0.3608	**<0.0001**	0.3822	**<0.0001**	0.2159	**0.0009**
	Adjusted ^2^	0.1614	**<0.0001**	0.0866	**0.0017**	0.2212	**<0.0001**	0.2181	**0.0002**
	Adjusted ^3^	0.1504	**<0.0001**	0.0901	**0.0010**	0.2026	**<0.0001**	0.0466	**0.0847**

^2^ Model adjusted for age, sex and total energy intakes, ^3^ Model adjusted for age, sex, total energy intakes and total BCAA, animal protein and red meat intakes. Significant values (*p* < 0.05) are presented in bold. Abbreviations: NW, normal weight; OW, overweight-obese; MS, metabolic syndrome; ACs, Acylcarnitines.

## Supplementary Materials

The following is available online at http://www.mdpi.com/2072-6643/11/1/173/s1, Table S1: Food grouping used in the dietary pattern analysis.
