# Supplementary material for: Associations Between Dietary Protein Sources, Plasma BCAA and Short-Chain Acylcarnitine Levels in Adults
Source: Nutrients. 2019 Jan 15;11(1):173. doi: 10.3390/nu11010173 (PMC6356602; doi:10.3390/nu11010173)
Supplement: Supplementary file 1 [file nutrients-11-00173-s001.pdf]

Supplementary TableS 1: Food grouping used in the dietary pattern analysis

Foods or food groups	Food items in the group
Red meat	Beef, veal, lamb, pork, game and horse; burger
Processed meat	Bacon, sausage, hot dogs, hot dogs on a stick, salami, bologna, ham, pepperoni, cretons, liver paste, terrine, head cheese
Organ meat	Liver or other organ meat such as heart, kidney and tongue
Fish and other seafood	Fish and sea food (all kinds); sushi
Poultry	Chicken and turkey with or without skin; chicken nuggets; hot chicken; club sandwich; chicken burger
Eggs	Eggs including scrambled, fried, omelets, hard-boiled eggs and quiche
Low-fat dairy products	Skim, 1% or 2%-fat milk (white or chocolate) and yogurt; frozen yogurt
High-fat dairy products	Whole milk (white or chocolate); cheese (all kinds); cream; ice cream; yogurt (>2%-fat); parmesan fondue; alfredo and white sauces
Legumes	Legumes including lentils, chickpeas, beans, peas soup; soy product including tofu, soy milk
Nuts	All nuts or seed and nuts butter
Refined grain products	White breads, bagels, pita, tortillas, English muffins, rice, pasta; muffins (home-made); couscous; pancakes; waffles; granola bar; refined cereal[2]
Whole grain products	Whole-wheat, whole-grain and other multigrain breads, bagels, tortillas, English muffins and pita; wheat or whole-grain pasta and brown rice; oatmeal and cream of wheat; whole-grain cereal[1]

[1] Cereal that contain ≤ 2.0g fiber/serving are in the refined grains group

[2] Cereal that contain > 2.0g fiber/serving are in the whole grains group
